# MMP-2, MMP-9, and TIMP-4 and Response to Aspirin in Diabetic and Nondiabetic Patients with Stable Coronary Artery Disease: A Pilot Study

**DOI:** 10.1155/2017/9352015

**Published:** 2017-07-10

**Authors:** Wiktor Kuliczkowski, Marek Radomski, Mariusz Gąsior, Joanna Urbaniak, Jacek Kaczmarski, Andrzej Mysiak, Marta Negrusz-Kawecka, Iwona Bil-Lula

**Affiliations:** ^1^Department and Clinic of Cardiology, Wroclaw Medical University, Borowska Street 213, 50-556 Wroclaw, Poland; ^2^Department of Pharmacology, College of Medicine, University of Saskatchewan, 107 Wiggins Road, Saskatoon, SK, Canada S7N 5E5; ^3^3rd Chair and Department of Cardiology, Silesian Center for Heart Diseases, M. Curie-Sklodowskiej Street 9, 41-800 Zabrze, Poland; ^4^Department of Laboratory Diagnostics, Lower Silesian Oncology Center in Wroclaw, Hirszfelda Street 12, 53-413 Wroclaw, Poland; ^5^Department of Clinical Chemistry, Wroclaw Medical University, Borowska Street 211A, 50-556 Wroclaw, Poland

## Abstract

**Background:**

High on-aspirin treatment platelets reactivity (HPR) is a significant problem in long-term secondary prevention of cardiovascular events. We hypothesize that imbalance between platelets MMPs/TIMPs results in cardiovascular disorders. We also explored whether chronically elevated blood glucose affects MMP-2/TIMP-4 release from platelets.

**Materials and Methods:**

Seventy patients with stable coronary artery disease, supplemented with aspirin, participated in this pilot study. The presence of HPR and/or diabetes mellitus was considered as the differentiating factor. Light aggregometry, impedance aggregometry, and ELISA tests for TXB2, MMP-2, MMP-9, and TIMP-4 were performed in serum, plasma, platelet-rich plasma, and platelets-poor plasma, as appropriate.

**Results:**

Aspirin-HPR did not affect plasma MMP-2, MMP-9, and TIMP-4. Arachidonic acid-induced aggregation of platelets from aspirin-HPR patients did not lead to increased release of MMP-2, MMP-9, and TIMP-4. Studying patients at the lowest TXB2 serum concentration quartile revealed that high concentration of plasma TIMP-4 and TIMP-4 negatively correlated with TXB2 and platelet aggregation. Diabetics showed an increased plasma MMP-2 as well as an increased MMP-2 in supernatants after platelet aggregation. However, diabetes mellitus did not affect MMP-9 and TIMP-4.

**Conclusion:**

Aspirin-HPR did not affect the translocation and release of MMPs and TIMP-4 from platelets. TIMP-4 may serve as a marker of TXA2-mediated platelet aggregation. Chronically elevated plasma glucose increases plasma MMP-2, and HPR potentiates this phenomenon.

## 1. Introduction

Diabetes mellitus (DM) is one of the major risk factors for the development of cardiovascular disease and a higher mortality [1]. It has been reported that patients with DM type 2 and no previous history of coronary artery disease (CAD) have similar risk for cardiac events to patients with prior myocardial infarction [2]. Apart from traditional risk factors for the development of cardiovascular events in diabetes subjects, nowadays a lot of attention is payed to nontraditional risk factors including haematological and thrombogenic factors. Atherothrombosis, defined as the formation of a thrombus on atherosclerotic plaque, is the leading cause of acute cardiovascular events [3]. Going further, it is well documented that hyperglycemia increases the expression and activity of matrix metalloproteinases (MMPs) in vascular macrophages and endothelial cells; hence it facilitates vascular remodeling and cardiovascular complications [4].

Matrix metalloproteinases are ubiquitous in the family of calcium-dependent zinc-containing endopeptidases that are mainly involved in the degradation and remodeling of extracellular matrix of the tissues. They are expressed at low level in normal adult tissue turnovers such as reproduction [5, 6], development [7], tissue repair [8], or immune response [9, 10] and are upregulated during pathological processes including inflammation [11], autoimmune diseases [12], neurogenerative disorders [13], tumor invasion and metastasis [14, 15], and heart injury [16]. Broad substrate specificities and strict regulation of their expression, activation, and inhibition levels contribute to maintenance of tissue homeostasis. The activity of MMPs is regulated mostly by the endogenous tissue inhibitors of metalloproteinases (TIMPs), which bind to the active site of MMPs and block access to extracellular matrix substrates [17, 18]. Besides the extracellular role of MMPs, several studies describe an intracellular action of MMPs in physiological and pathological states [19–21] in which both MMP-2 and MMP-9 as well as TIMP-4 have been identified in platelets [22]. During aggregation, MMP-2 and MMP-9 are translocated from the cytosol to the platelet surface [22, 23] where MMP-2 remains in close association with platelet membrane adhesion receptors affecting their activation and the aggregatory response of platelets [24]; MMP-9 shows an opposite antiaggregatory activity [23, 25]. It was also reported that TIMP-4 is colocalized with MMP-2 in resting platelets and is released from platelets upon aggregation [26]. On the basis of this evidence we hypothesize that the dissociation of TIMP-4 from TIMP-4-MMP-2 complex and release of this proteins into extracellular space may regulate platelets aggregation.

Aspirin (acetylsalicylic acid) inhibits platelet aggregation by irreversible inactivation of cyclooxygenase enzyme (COX-1), which is involved in prostaglandins and thromboxane A_2_ synthesis [27, 28]. As a factor of decreased risk of cardiovascular incidents, it is widely used in clinical practice during coronary interventions and in long-term secondary prevention of cardiovascular and cerebrovascular events. However, in some patients, a high on-aspirin treatment platelet reactivity (HPR), referred to as a higher than expected platelet reactivity in patients under antiplatelet therapy, is observed. The limited degree of inhibition of platelet function is associated with poor cardiovascular outcomes and might be of clinical value for identifying patients with high risk of recurrent vascular events who may benefit from intensified antiplatelet therapy [29]. While HPR has been widely described in many papers, a precise mechanism has not been clearly explained. There are some contrary results showing an influence of aspirin on MMP-2/TIMP pathways in platelets. Falcinelli et al. (2007) and others showed that treatment with aspirin did not affect the translocation and release of MMP-2 from platelets [30, 31], but Hua et al. (2009) reported that aspirin decreased the expression and release of MMP-2 and MMP-9 from monocytes [32]. Others showed that MMP-9 can influence the action of aspirin through modification of the TXA_2_ pathway [23] and that aspirin can influence MMP-2 and MMP-9 production in monocytes [32] and megakaryocytes [33, 34]. Based on these discrepancies, the main aim of the current pilot study was to explore if MMPs/TIMP-4 interactions in platelets or plasma are associated with the response to aspirin in patients with diabetes and stable coronary artery disease and whether chronically elevated blood glucose affects MMP-2/TIMP-4 release from platelets.

## 2. Material and Methods

### 2.1. Study Group and Clinical Material

Seventy patients with stable coronary artery disease participated in this study. Clinical characteristics of study participants are presented in Table 1. All participants were recruited by the Department and Clinic of Cardiology, Wroclaw Medical University in Wroclaw. Written informed consent was obtained for the collection of blood samples. The study was approved by the local Ethics Committee of the Medical University of Silesia. Study subjects were informed in detail about the purpose and the principles of this study. 30 ml of citrate anticoagulated whole blood (1 + 9, v : v) was collected for further analysis.

### 2.2. Criteria for Classification

Inclusion criteria for the study included stable coronary artery disease and aspirin use in a dose of 75 mg per day for at least 7 days preceding study inclusion. Exclusion criteria incorporated intake of antiplatelet drugs other than aspirin during two weeks before study inclusion, percutaneous coronary intervention (PCI) or coronary artery bypass grafting (CABG) up to 3 months before study inclusion, current bleeding and anemia, and platelets count in whole blood below 150,000/mm^3^ or above 450,000/mm^3^.

### 2.3. Light Aggregometry (LTA)

Light aggregometry was performed with the use of Chronolog “560 Ca” aggregometer (Havertown, USA). Briefly, sodium citrate anticoagulated whole blood (0.109 M) was centrifuged at 100*g* for 15 minutes without braking to obtain platelet reach plasma (PRP). Half of the PRP volume was centrifuged again (20 minutes at 2400*g*) to obtain platelet poor plasma (PPP) which served as a blank. The platelet count in PRP was adjusted to 300,000/mm^3^. Platelets aggregation was measured after the addition of arachidonic acid as an agonist (Chronolog, Havertown, USA) with a final concentration of 0.5 mg/ml. Maximum platelet aggregation during a 5-minute interval was assessed. The range of values for LTA was 0–100%. Results were given in percentage of light transmittance. Every aggregation measurement was performed in duplicate with the mean subtraction. If 10% difference between measurements and the mean appeared, additional aggregation measurements were performed.

### 2.4. Impedance Aggregometry (IMA)

Multiplate Aggregometer was used (Roche, France) for impedance in the whole blood aggregation measurement. Blood was collected into tubes containing hirudin (25 *μ*g/ml) (Sarstedt, Germany). Arachidonic acid in final concentration of 0.5 mM (Roche, France) was used as an agonist. Results were given as areas under aggregation curves in arbitral units (AU). Every aggregation measurement was performed in duplicate, and if the difference between measurements was above 10%, another two measurements were performed.

### 2.5. TXB2 Concentration in Serum

Blood was collected in dry tubes and then heated at 37°C for 60 minutes. Serum samples were collected by centrifugation. The concentration of serum TXB2 was measured by ELISA test (R&D, USA).

### 2.6. MMP-2, MMP-9, and TIMP-4 in Plasma and Supernatant of PRP

Sodium citrate anticoagulated blood was collected on ice and centrifuged (1000 ×g, 20 min, 4°C) immediately after collection and separated plasma samples were used for the assessment of metalloproteinases and their inhibitor concentrations. Commercially available ELISA tests for MMP-2 (Total MMP-2 Quantikine ELISA), MMP-9 (Human MMP-9 Quantikine ELISA), and TIMP-4 (Human TIMP-4 Quantikine ELISA) (R&D, USA) were used. Total MMP-2 including active MMP-2, pro-MMP-2, and TIMP complexed matrix metalloproteinase 2 as well as active and proenzyme of MMP-9 concentrations were measured. The minimum detectable dose (MDD) was on average 0.033 ng/mL for MMP-2, less than 0.156 ng/mL for MMP-9, and on average 4.91 pg/mL for TIMP-4. Total MMP-2 Quantikine ELISA assay recognized recombinant MMP-2 and natural human, mouse, rat, porcine, and canine active MMP-2, pro-MMP-2, and TIMP complexed MMP-2. Human MMP-9 Quantikine ELISA test was able to measure natural and recombinant 92 kDa pro-MMP-9 and the 82 kDa active MMP-9. It did not measure the 65 kDa form of MMP-9. In turn, Human TIMP-4 Quantikine ELISA recognized natural and recombinant human TIMP-4.

To study platelets release of MMP-2, MMP-9, and TIMP-4 into extracellular space, their concentration was also measured in supernatants of PRP after platelet aggregation (see optical aggregometry above). Additionally, to study an influence of aspirin on MMPs and TIMP-4 release from platelets, the optical aggregometry with arachidonic acid after 5-minute incubation of PRP with aspirin 100 *μ*g/ml (Laspal, Polfa, Poland) was performed. Supernatants after aggregation and PPP used as a blank for LTA (obtained by blood centrifugation at room temperature) were also used to measure MMPs/TIMP-4 concentrations.

### 2.7. High On-Aspirin Treatment Platelet Reactivity (HPR)

Aspirin-high on-treatment platelets reactivity was defined to be present when in vitro platelet reactivity (assessed by the use of single laboratory test) was not properly blocked despite the use of oral antiplatelet drugs, according to established criteria [28]. Five different criteria were used to determine HPR. HPR in light aggregometry (optical aggregation) was present if maximal aggregation exceeded 20% or in impedance method was above 30 AU (0 Ω). TXB2 level higher than 3.1 ng/ml was also considered to be HPR. Additionally, LTA and TXB2 level were divided into quartiles and correlated with MMPs/TIMP concentrations.

### 2.8. Statistics

Statistica 12 software (StatSoft, USA) was used for data analysis. Results were expressed as mean ± SEM or median (interquartile range). Data that showed a right-skewed distribution but met the remaining criteria for the normal distribution was transformed logarithmically and analyzed by relevant tests. Shapiro, one-way or two-way ANOVA and Mann-Whitney *U* tests were used as appropriate. To confirm the homogeneity of compared groups *χ*^2^ test with Yates correction has been used. Spearman's or Kendall's rank correlation was used to assess the correlations.

## 3. Results

### 3.1. Plasma Concentration of MMP-2, MMP-9, and TIMP-4 in Patients with High On-Aspirin Treatment Platelets Reactivity (HPR)

**Figure 1:**
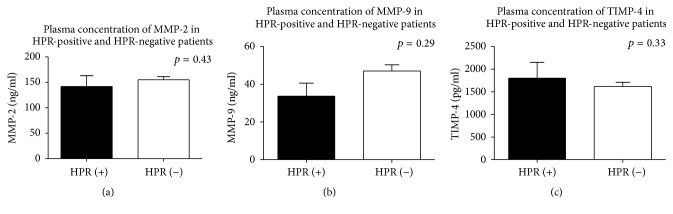
An influence of HPR on plasma concentration of MMP-2 (a), MMP-9 (b), and TIMP-4 (c). Mean ± SEM classification of HPR on the basis of LTA Amax > 20%. HPR-high on-aspirin treatment platelets reactivity; LTA: light transmittance aggregometry; MMP: matrix metalloproteinase; TIMP-4: tissue inhibitor of MMPs.

HPR was tested according to established criteria by use of light transmittance and multielectrode aggregometry or by measurement of serum TXB2. Data showed that plasma concentrations of MMP-2, MMP-9, and TIMP-4 were similar both in HPR patients and their counterparts (Figure 1). Since concordance among different tests in the identification of patients with HPR is limited [35], we have compared the concentrations of matrix metalloproteinases and their inhibitor in HPR patients classified by different methods. Despite testing different criteria for aspirin-HPR (cut off point >15%, >20%, >30 AU, >3.1 ng/ml, and >5.8 ng/ml, as appropriate), we did not show a significant difference in MMP-2, MMP-9, and TIMP-4 concentrations in plasma of aspirin responding and aspirin-HPR patients (Table 2).

### 3.2. MMP-2, MMP-9, and TIMP-4 Release from Aggregating Platelets Obtained from HPR-Positive and HPR-Negative Patients

**Figure 2:**
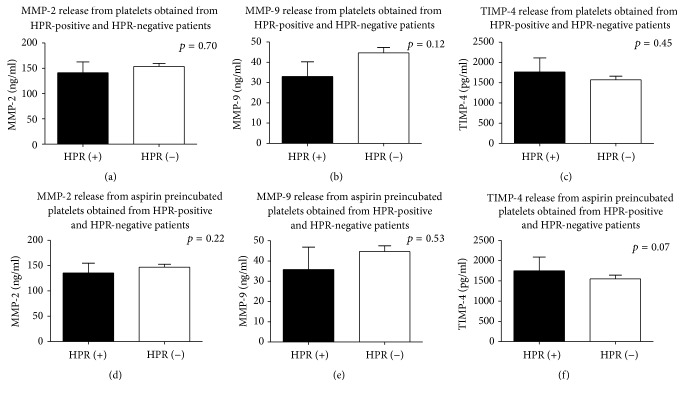
An influence of HPR on platelets release of MMP-2, MMP-9, and TIMP-4 without (a–c) or with (d–f) pretreatment with parenteral form of aspirin. Mean ± SEM classification of HPR on the basis of LTA Amax > 20%. HPR-high on-aspirin treatment platelets reactivity; LTA: light transmittance aggregometry; MMP: matrix metalloproteinase; TIMP-4: tissue inhibitor of MMPs.

To determine whether insufficient platelet inhibition in patients with high on-aspirin treatment platelets reactivity affects platelets MMPs/TIMP-4 pathway, the concentration of MMP-2, MMP-9, and TIMP-4 in supernatants obtained due to platelets aggregation was determined. We reported that arachidonic acid-induced aggregation of platelets from aspirin-HPR patients did not reveal an increased release of MMP-2, MMP-9, and TIMP-4 in comparison to patients with sufficient platelet inhibition (Table 2, Figures 2(a)–2(c)).

Additionally, to study a direct influence of aspirin on MMPs and TIMP-4 release from platelets, an optical aggregometry with arachidonic acid after 5-minute incubation of platelet-rich plasma with aspirin was performed. We showed that mean concentrations of MMP-2, MMP-9, and TIMP-4 in supernatants after aggregation of aspirin preincubated platelets were similar in patients with aspirin-HPR and in patients with properly inhibited platelets (Table 2, Figures 2(d)–2(f)).

We also measured the concentration of MMPs/TIMPs in PPP (used as a blank for LTA), obtained by blood centrifugation at room temperature. There was no significant difference (*p* > 0.05) in MMPs/TIMPs concentration between following samples: PPP from LTA obtained at room temperature versus plasma obtained from cold centrifugation versus PRP supernatants after aggregation versus PRP supernatants after aggregation with aspirin preincubation.

### 3.3. An Influence of TIMP-4 on Production of TXB2 and Platelets Aggregation

**Figure 3:**
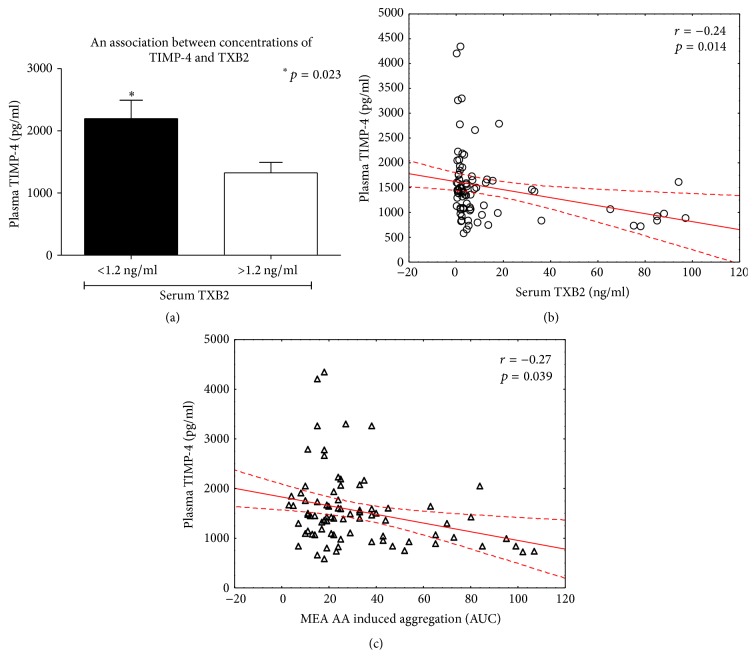
An association of TIMP-4 with TXB2 (a-b) and platelets aggregation (c). Mean ± SEM; platelets aggregation tested by MEA. MEA: multielectrode aggregometry; TXB2: thromboxane B2; TIMP-4: tissue inhibitor of MMP.

We found that patients at the lowest TXB2 serum concentration quartile (below 1.2 ng/ml) had higher antiaggregatory TIMP-4 plasma concentration in comparison to patients with higher TXB2 concentration (2195.8 ± 942.3 versus. 1325.0 ± 526.5; *p* = 0.023) (Figure 3(a)). Plasma TIMP-4 negatively correlated with TXB2 (*r* = −0.24, *p* = 0.014) (Figure 3(b)) and platelets aggregation (*r* = −0.27, *p* = 0.039) (Figure 3(c)).

### 3.4. MMPs and TIMP-4 in Patients with or without Diabetes

**Figure 4:**
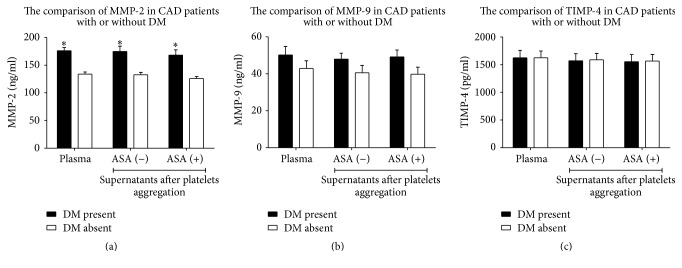
The comparison of MMP-2 (a), MMP-9 (b), and TIMP-4 (c) concentrations in plasma and supernatants after platelets aggregation in patients with or without diabetes mellitus (DM). Mean ± SEM. ASA: aspirin; CAD: coronary artery disease; DM: diabetes mellitus; MMP-2: matrix metalloproteinase 2; MMP-9: matrix metalloproteinase 9; TIMP-4: tissue inhibitor of MMPs; ^*∗*^*p* < 0.001.

DM-positive and DM-negative patients were verified in respect of MMP-2, MMP-9, and TIMP-4 concentration in plasma and supernatants after platelets aggregation. Diabetics with CAD showed an increased plasma concentration of MMP-2 as well as an increased MMP-2 in supernatants after platelets aggregation in comparison to CAD patients without diabetes (Table 3, Figure 4(a)). However, DM had no influence on MMP-9 and TIMP-4 concentrations in plasma and their release from platelets during aggregation (Table 3, Figures 4(b) and 4(c)).

Interestingly, although the presence of HPR had no influence on plasma level of MMP-2 as well as its release from activated platelets (as indicated above), diabetic subjects with HPR (meeting MEA criteria) showed an increased plasma MMP-2 in comparison to diabetics without HPR (213.6  ±  76.1 versus 164.8 ± 54.6, borderline significance *p* = 0.052).

## 4. Discussion

Acetylsalicylic acid is widely used in clinical practice during coronary interventions and in long-term secondary prevention of cardiovascular and cerebrovascular events. Unfortunately, in some patients, higher than expected platelet reactivity during antiplatelet therapy (a high on-treatment platelet reactivity, HPR) is observed. The limited inhibition of platelet function due to incomplete inactivation of cyclooxygenase 1 enzyme (COX-1) [27, 28] is associated with poor cardiovascular outcomes [29]. Sawicki et al. (1997) showed that MMP-2 is released from platelets during platelets stimulation and facilitates their aggregation by non-ADP and non-TXA2 pathway [25, 26]; building upon this finding, some contrary results describing an influence of aspirin on MMPs/TIMP pathway have been published. Falcinelli et al. (2007) and others showed that treatment with aspirin did not affect the translocation and release of MMP-2 from platelets into plasma [30, 31], but Hua et al. (2009) reported that aspirin decreases an expression and release of MMP-2 and MMP-9 from monocytes [32]. Others showed that MMP-9 can influence the action of aspirin through modification of the TXA_2_ pathway [23] and that aspirin can influence MMP-2 and MMP-9 production in monocytes [32] and megakaryocytes [33, 34].

In our study we observed that plasma concentration of MMP-2 in patients with high on-aspirin treatment platelets reactivity was similar to those with proper response to aspirin (HPR-negative). This suggests that the concentration of plasma MMP-2 was not associated with an increased aggregation of platelets in HPR-positive patients. Following the previous hypothesis that MMP-2 is released from platelets during their aggregation and potentiates platelets aggregation [25], we explored whether decreased susceptibility of platelets to aspirin treatment affects release of MMP-2 in vivo. Data showed that platelets with lower response to aspirin released as much MMP-2 as platelets with total inhibition of COX1. This means that inhibition of COX1/COX2 in platelets of patients treated with aspirin has little impact on MMP-2 release. Moreover, indirect inhibition of platelets with aspirin before its stimulation with arachidonic acid (AA) did not show a significant effect on MMP-2 release. Therefore, other COX-mediated mechanisms associated with platelets release of metalloproteinases likely exist, or platelets excretion of MMP-2 has little or no impact on total plasma concentration of MMP-2 in vivo. Falcinelli et al. (2007) also showed that administration of aspirin did not significantly affect the surface expression and release of MMP-2 from platelets activated by vessel damage or TRAP [30]. Although previous in vitro studies showed that MMP-2 is released by platelets upon activation, nonphysiological stimuli (very high concentrations of thrombin) were used and were not affected from physiological factors regulating platelets activation in vivo [25]. Because platelets activated with AA in our in vivo study did not increase the total amount of plasma MMP-2, this may also suggest that plasma MMP-2 is mainly subjected to MMP-2 released from other blood or tissue cells.

In the previous studies, it was shown that platelet release of MMP-2 was triggered by collagen induced aggregation [24] while its release upon thrombin was controversial [36]. This is the first report focusing on MMP-2 release from platelets activated by arachidonic acid (AA). Taking into account the fact that there was no difference in the platelets' release of MMP-2 in our study, it is very likely that arachidonic acid affects platelets' excretion of MMP-2 independently of COX-1/COX-2 inhibition by aspirin. A study by Hermann et al. (2001) supports this hypothesis by showing that translocation of cytosol proteins into platelets' surface during aggregation is resistant to aspirin inhibition [37].

MMP-9 is another platelets-derived matrix metalloproteinase [38]. The antiaggregatory role of MMP-9 based on TXA2 dependent pathway and independent of stimulator of aggregation has been well documented [23, 38, 39]. Both MMP-9 and MMP-2 were shown to be released from platelets into coronary circulation during acute coronary syndrome, showing the potential association of MMPs and the development of acute coronary syndrome [30]. Although the potential for acetylation of serine at the catalytic site of COX-1/COX-2 by aspirin inhibits the release of matrix metalloproteinases from platelets [40], we did not show an influence of aspirin on MMP-9 excretion and on its antiaggregatory function in subjects with proper or insufficient responsiveness to aspirin. Moreover, plasma concentration of MMP-9 was slightly decreased in patients with high on-aspirin treatment platelets reactivity, suggesting that the response of platelets to aspirin treatment in HPR patients is opposite to our previous hypothesis. Potentially, HPR should lead to an increased expression and release of platelet MMPs due to incomplete inhibition of COX-1/COX-2 pathway. Here, we observed slightly decreased plasma concentration of MMP-9 in HPR-positive patients and no MMP-9 release from platelets upon stimulation with arachidonic acid. Previous studies reported contrary data about the release of MMP-9 upon stimulation with collagen, thrombin, and TLR2 agonist Pam3CSK4 [36, 39]. The same authors suggested that presence of MMP-9 in peripheral blood was attributed mainly to neutrophil release [36]. Some investigators are even skeptical of the presence of MMP-9 in platelets [41, 42].

Higher mortality due to acute myocardium infarction is observed in diabetic patients than in nondiabetic subjects [2]. An increased propensity of atherosclerotic plaques to ulceration and thrombosis in diabetics enhanced a risk of fatal outcome [43]. Regulation of MMPs in DM has been widely investigated. Hyperglycemia increases an expression and activity of MMP-2 and MMP-9 in aortic smooth muscle cells, vascular tissue, and plasma [4, 44], affecting metabolism of the extracellular matrix. Studies suggest that hyperglycemia increases oxidative stress in various cells, leading to an activation of COX-2 which in turn induces a biosynthesis of MMP-2 and MMP-9 [45, 46]. Schulze et al. (2003) reported that imbalance between MMPs and TIMPs results in enhanced MMP activity and then in several cardiovascular disorders [47]. Because MMPs participate in rupture of atherosclerotic plaques [48], we decided to check whether chronically elevated plasma glucose increases the expression and release of MMP-2 and MMP-9, hence accelerating the risk for acute coronary syndromes. We showed that diabetic patients with coronary artery disease presented significantly higher plasma levels of MMP-2, but not MMP-9, than nondiabetic counterparts. We also exclude that other factors such as arterial hypertension, history of myocardial infarction, and hypercholesterolemia had an influence on MMP-2 level in DM patients (Table 1). Our results are consistent with previous reports showing that hyperglycemia is associated with increased plasma concentration of MMP-2 [49–51]. We suspect that nonsignificant increase of MMP-9 resulted from the small number of tested subjects. However, data from Baugh et al. (2003) revealed no significant difference in MMP-9 production in DM [52], and data from Bhatt and Veeranjaneyulu (2014) indicated that MMP-2 level was highly elevated in comparison to MMP-9 [53]. Matrix metalloproteinases are regarded to be the key molecules in inflammation [10, 54]. They are implicated in the accumulation of inflammatory cells, healing of tissue injury, and remodeling processes [55, 56]. Taking into account the fact that diabetes mellitus is a chronic inflammatory disease, an induction of proinflammatory factors leads in consequence to recruitment of monocytes, macrophages, and granulocytes, which are a rich source of MMPs. Since all participants of our study group had coronary artery disease and only those suffering from DM had elevated plasma MMP-2, an important observation is that DM was a strong determinant of increased plasma MMPs. However, taking into account the fact that plasma concentration of MMP-2 was also elevated in the group of diabetic subjects with aspirin-HPR with respect to diabetics without aspirin-HPR and high on-aspirin treatment platelets reactivity did not increase plasma MMP-2 irrespective to diabetes mellitus, this suggests that coincidence of both DM and HPR may affect plasma level of MMP-2.

Physiologically, matrix metalloproteinase activity in the extracellular matrix is regulated by the family of tissue inhibitors of matrix metalloproteinases (TIMPs). Because gelatinases bind TIMPs to form a tightly bound 1 : 1 molar stoichiometric complex [57], an increased expression of MMP-2 should be accompanied by enhanced production of TIMPs [50]. Since TIMP-1 and TIMP-2 may also be present in platelets [58], it was shown that TIMP-4 is the major intraplatelet inhibitor of MMPs [26]. Moreover, it was demonstrated that TIMP-4 and MMP-2 colocalized in resting platelets and upon aggregation by aggregating agents such as collagen and thrombin MMP-2 is translocated to the platelet surface and TIMP-4 is released to plasma. Hence, the dissociation of TIMP-4 from its complex with MMP-2 may facilitate the interactions of MMP-2 with its receptors and stimulate aggregation [24]. However, an increased plasma concentration of MMP-2 in diabetics of our study was not accompanied by increased plasma concentration of TIMP-4. This suggests that hyperglycemia affects the physiological production of TIMP-4 following increased MMP-2 synthesis. Radomski et al. (2002) reported that TIMP-4 is colocalized with MMP-2 in resting platelets and is released from platelets upon aggregation [26]. Hence we hypothesized that the dissociation of TIMP-4 from TIMP-4-MMP-2 complex may stimulate platelets aggregation due to interactions of MMP-2 with its receptors on platelets. Also in this case our study did not confirm that dissociation of TIMP-4 and MMP-2 from their complex led to enhanced release of MMP-2/TIMP. It is most likely that enhanced concentrations of plasma MMP-2 in DM subjects were caused by stimulation of cells other than platelets.

Although we did not notice a significant in vitro release of TIMP-4 from platelets after stimulation with arachidonic acid and there was no association between aspirin-HPR and plasma level of TIMP-4, we showed that plasma concentration of TIMP-4 was the highest in patients with the lowest TXB2 level. Together with the negative correlation between TIMP-4, TXB2, and platelets aggregation, this could imply TIMP-4 as a marker of low on-treatment platelet reactivity.

In conclusion, our pilot study observed that administration of aspirin for one week before blood collection from aspirin-HPR-positive and aspirin-HPR-negative subjects was not associated with translocation and release of both matrix metalloproteinases and its selective inhibitor from platelets into plasma. Also direct treatment of platelets with ASA did not affect platelets release of MMPs/TIMP-4 during activation and aggregation induced by arachidonic acid. The contribution of MMP-2, MMP-9, and TIMP-4 in the regulation of platelets function in patients with high on-aspirin treatment platelets reactivity is negligible and decreased plasma concentration of TIMP-4 may serve as a marker of TXA2-mediated platelets aggregation. It is also worth noting that chronically elevated plasma glucose increases plasma concentration of MMP-2, and aspirin-HPR potentiates this phenomenon. For this reason, an inhibition of MMP-2 with selective inhibitors should be considered for the prevention of DM-induced cardiovascular complications.

## Figures and Tables

**Table 1:** Clinical characteristics of the study population.

Clinical parameter	Number of patients (%)		Statistical significance
	CAD, diabetes group	CAD, no-diabetes group	
Total number of patients	35 (50)	35 (50)	
Age (years), mean ± SEM	62.7 ± 1.5	60.1 ± 1.6	NS
Sex			
Men	27 (77.1)	28 (80.0)	NS
Women	8 (22.9)	7 (20.0)	NS
Clinical characteristics			
Arterial hypertension	29 (82.8)	28 (80.0)	NS
Current tobacco use	10 (28.5)	15 (42.8)	NS
History of myocardial infarction	21 (60.0)	13 (37.1)	NS
History of PCI/CABG	18 (51.4)/7 (20.0)	10 (28.6)/3 (8.6)	NS
History of stroke or TIA	3 (8.5)	2 (5.7)	NS
Kidney insufficiency (GFR < 60 ml/min/m^2^),	10 (28.5)	5 (14.2)	NS
Hypercholesterolemia	35 (100)	30 (85.7)	NS
HbA1C, mean ± SD	6.8 ± 3.0	NA	NA
Drug administration			
Beta-blocker	35 (100)	32 (91.4)	NS
Calcium channel blockers	20 (57.1)	22 (62.8)	NS
ACE-I	30 (85.7)	31 (88.5)	NS
ARB	5 (14.2)	10 (28.5)	NS
Statins	35 (100)	35 (100)	NS
Oral antidiabetic drugs	30 (85)	NA	NA
Insulin	21 (65)	NA	NA
HPR criteria			
LTA (Amax > 20%)^*∗*^	2 (5.7)	1 (2.8)	NS
LTA (Amax > 15%) (highest quartile)^*∗*^	8 (22.8)	4 (11.4)	NS
MEA (AspiTEST > 30 AU)	10 (28.6)	9 (25.7)	NS
TXB2 > 3.1 ng/ml	15 (42.9)	11 (31.4)	NS
TXB2 > 5.8 ng/ml (highest quartile)	9 (25.7)	8 (22.9)	NS

*Notes*. ACE-I: angiotensin converting enzyme inhibitor; ARB: angiotensin receptor blocker; CABG: coronary artery bypass grafting; GFR: glomerular filtration rate; HbA1C: glycated hemoglobin A1C; LTA: light aggregometry; MEA: multielectrode aggregometry; NA: not analyzed; NS: not statistically significant; SD: standard deviation; PCI: percutaneous coronary intervention; TXB2: thromboxane B2; TIA: transient ischemic attack; ^*∗*^a range of values for LTA was 0–100%.

**Table 2:** MMP-2, MMP-9, and TIMP-4 concentrations in aspirin good responders and aspirin-HPR patients (according to different criteria).

MMPs/TIMP-4	LTA (Amax > 20%)		*p* value	LTA (Amax > 15%)		*p* value	MEA		*p* value	TXB2 > 3.1 ng/ml		*p* value	TXB2 > 5.8 ng/ml		*p* value
				(highest quartile)			(AspiTEST > 30 AU)						(highest quartile)		
	HPR +	HPR −		HPR +	HPR −		HPR +	HPR −		HPR +	HPR −		HPR +	HPR −	
	(*n* = 3)	(*n* = 67)		(*n* = 12)	(*n* = 58)		(*n* = 19)	(*n* = 51)		(*n* = 26)	(*n* = 44)		(*n* = 17)	(*n* = 53)	
Plasma level															

MMP-2 (ng/ml)	141.8 ± 21.2	155.1 ± 6.38	0.43	153.4 ± 11.3	154.8 ± 7.0	0.09	168.6 ± 15.1	149.9 ± 6.1	0.16	161.9 ± 12.4	149.7 ± 6.5	0.08	137.6 ± 7.9	160.6 ± 7.8	0.11
MMP-9 (ng/ml)	33.7 ± 6.8	47.0 ± 3.3	0.29	49.1 ± 5.4	45.9 ± 3.6	0.17	53.4 ± 6.6	43.6 ± 3.5	0.55	50.2 ± 4.8	41.4 ± 3.8	0.14	51.5 ± 7.0	42.6 ± 3.1	0.78
TIMP-4 (pg/ml)	1796.3 ± 354.1	1614.1 ± 94.4	0.33	1757.5 ± 222.8	1593.9 ± 99.7	0.44	1502.9 ± 136.8	1676.9 ± 114.1	0.17	1379.7 ± 106.7	1726.2 ± 127.8	0.87	1466.5 ± 140.0	1630.5 ± 112.2	0.06

Supernatant of PRP after LTA induced by AA															

MMP-2 (ng/ml)	141.1 ± 21.3	153.6 ± 6.0	0.70	153.3 ± 10.0	153.1 ± 6.7	0.06	166.4 ± 14.4	148.0 ± 5.9	0.90	157.1 ± 11.7	149.7 ± 6.3	0.15	134.2 ± 8.4	159.2 ± 7.3	0.13
MMP-9 (ng/ml)	33.0 ± 7.2	44.7 ± 2.67	0.12	48.3 ± 5.8	43.3 ± 2.8	0.34	43.3 ± 4.5	43.9 ± 3.1	0.14	43.9 ± 3.3	43.8 ± 3.5	0.78	42.8 ±.4.5	44.2 ± 3.0	0.62
TIMP-4 (pg/ml)	1762.6 ± 346.2	1568.9 ± 89.9	0.45	1694.0 ± 207.7	1553.0 ± 95.4	0.09	1465.3 ± 128.7	1629.6 ± 108.8	0.07	1364.5 ± 101.8	1662.4 ± 122.4	0.80	1440.9 ± 136.7	1579.4 ± 106.5	0.76

Supernatant after 5 minutes of aspirin incubation and subsequent LTA induced by AA															

MMP-2 (ng/ml)	135.6 ± 19.2	146.9 ± 5.9	0.22	148.1 ± 10.9	146.0 ± 6.5	0.08	157.7 ± 12.5	142.9 ± 6.2	0.08	151.6 ± 11.8	142.8 ± 6.4	0.08	129.2 ± 7.4	152.4 ± 7.2	0.34
MMP-9 (ng/ml)	35.8 ± 6.5	44.7 ± 2.8	0.53	51.7 ± 7.8	42.8 ± 2.8	0.41	40.6 ± 3.7	45.4 ± 3.4	0.36	45.1 ± 2.8	42.6 ± 3.5	0.78	44.2 ± 4.0	43.4 ± 3.0	0.08
TIMP-4 (pg/ml)	1748.0 ± 340.3	1548.4 ± 91.4	0.07	1669.4 ± 208.9	1533.7 ± 97.7	0.76	1459.3 ± 130.8	1604.1 ± 110.7	0.73	1360.7 ± 103.2	1632.7 ± 124.8	0.67	1439.7 ± 140.6	1553.7 ± 107.8	0.16

*Notes*. AA: arachidonic acid; AspiTEST: arachidonic acid-induced aggregation in MEA; LTA: light transmittance aggregometry; MEA: multielectrode aggregometry; MMP-2: matrix metalloproteinase-2; MMP-9: matrix metalloproteinase-9; TIMP-4: tissue inhibitor of matrix metalloproteinase-4; TXB2: thromboxane B2; HPR: high on treatment platelet reactivity; mean ± SEM.

**Table 3:** MMP-2, MMP-9, and TIMP-4 level in diabetes and nondiabetes subjects.

	DM present and CAD present	DM absent and CAD present	Statistical significance
	(*n* = 35)	(*n* = 35)	
Plasma level			

MMP-2 (ng/ml)	176.3 ± 5.5	134.0 ± 3.8	*p* < 0.001
MMP-9 (ng/ml)	50.2 ± 4.6	42.9 ± 4.2	NS
TIMP-4 (pg/ml)	1621.9 ± 134.4	1622 ± 123.4	NS

Supernatant after LTA induced by AA			

MMP-2 (ng/ml)	174.7 ± 9.8	132.7 ± 4.0	*p* < 0.001
MMP-9 (ng/ml)	47.9 ± 3.2	40.6 ± 3.9	NS
TIMP-4 (pg/ml)	1569.8 ± 127.4	1584.1 ± 117.9	NS

Supernatant after 5 minutes of aspirin incubation and subsequent LTA induced by AA			

MMP-2 (ng/ml)	168.2 ± 9.5	125.9 ± 3.7	*p* < 0.0001
MMP-9 (ng/ml)	49.2 ± 3.6	39.8 ± 3.8	NS
TIMP-4 (pg/ml)	1550.2 ± 129.4	1563.3 ± 119.8	NS

*Notes*. AA: arachidonic acid, CAD: coronary artery disease, DM: diabetes mellitus, HPR: high on-treatment platelet reactivity, LTA: light transmittance aggregometry, MMP-2: matrix metalloproteinase-2, MMP-9: matrix metalloproteinase-9, MEA: multielectrode aggregometry, TIMP-4: tissue inhibitor of matrix metalloproteinase-4, NS: statistically not significant, TXB2: thromboxane B2; mean ± SEM.

## Additional Points

*Study Limitations*. The study group is rather small but the results, especially those obtained in vitro, are consistent. Data represents a pilot study. In previous reports, MMPs and TIMP-4 were shown to be released upon platelet activation with collagen and to some extent thrombin. We used arachidonic acid as an agonist of aggregation, because (a) it is widely accepted to monitor aspirin response [28], (b) MMPs/TIMP release from platelets was not tested after arachidonic acid stimulation so far, and (c) this agonist was chosen to assess aspirin effect and possible MMPs/TIMP interaction. We measured MMPs/TIMP plasma concentration, not activity, because the use of zymography which allows for measurement of activity of both active and nonactive forms of enzymes (proenzymes and enzymes) seems doubtful.
